# Sorting Live Stem Cells Based on *Sox2* mRNA Expression

**DOI:** 10.1371/journal.pone.0049874

**Published:** 2012-11-27

**Authors:** Hans M. Larsson, Seung Tae Lee, Marta Roccio, Diana Velluto, Matthias P. Lutolf, Peter Frey, Jeffrey A. Hubbell

**Affiliations:** 1 Laboratory for Regenerative Medicine and Pharmacobiology, Institute for Bioengineering, School of Life Sciences and School of Engineering, Ecole Polytechnique Fédérale de Lausanne, Lausanne, Switzerland; 2 Laboratory of Stem Cell Biomodulation, Department of Animal Biotechnology, Kangwon National University, Chuncheon, Korea; 3 Laboratory of Stem Cell Bioengineering, Institute for Bioengineering, School of Life Sciences, Ecole Polytechnique Fédérale de Lausanne, Lausanne, Switzerland; 4 School of Biological and Chemical Sciences, Queen Mary, University of London, London, United Kingdom; 5 Laboratory of Experimental Pediatric Urology, Department of Pediatric Urology, Centre Hospitalier Universitaire Vaudois, Lausanne, Switzerland; City of Hope National Medical Center and Beckman Research Institute, United States of America

## Abstract

While cell sorting usually relies on cell-surface protein markers, molecular beacons (MBs) offer the potential to sort cells based on the presence of any expressed mRNA and in principle could be extremely useful to sort rare cell populations from primary isolates. We show here how stem cells can be purified from mixed cell populations by sorting based on MBs. Specifically, we designed molecular beacons targeting *Sox2,* a well-known stem cell marker for murine embryonic (mES) and neural stem cells (NSC). One of our designed molecular beacons displayed an increase in fluorescence compared to a nonspecific molecular beacon both in vitro and in vivo when tested in mES and NSCs. We sorted *Sox2*-MB*^+^*SSEA1^+^ cells from a mixed population of 4-day retinoic acid-treated mES cells and effectively isolated live undifferentiated stem cells. Additionally, *Sox2*-MB*^+^* cells isolated from primary mouse brains were sorted and generated neurospheres with higher efficiency than *Sox2*-MB*^−^* cells. These results demonstrate the utility of MBs for stem cell sorting in an mRNA-specific manner.

## Introduction

Fluorescence-activated cell sorting (FACS) of live cells is typically performed using antibodies that bind to proteins present on the cell surface or using intracellular co-expressed fluorescent reporter proteins. For characterization of embryonic stem cells and induced pluripotent stem cells, the expression of transcription factors, such as *Oct4*, *Nanog* and *Sox2*, is at present the most meaningful indication of stemness, yet the presence of these intracellular proteins cannot be detected in living cells without the use of co-expressed fluorescent reporters [1], [2], [3]. It would be useful be able to omit the insertion of fluorescence reporters and instead sort based on transcription factor expression in a manner that is independent of genetic modification.

**Figure 1:**
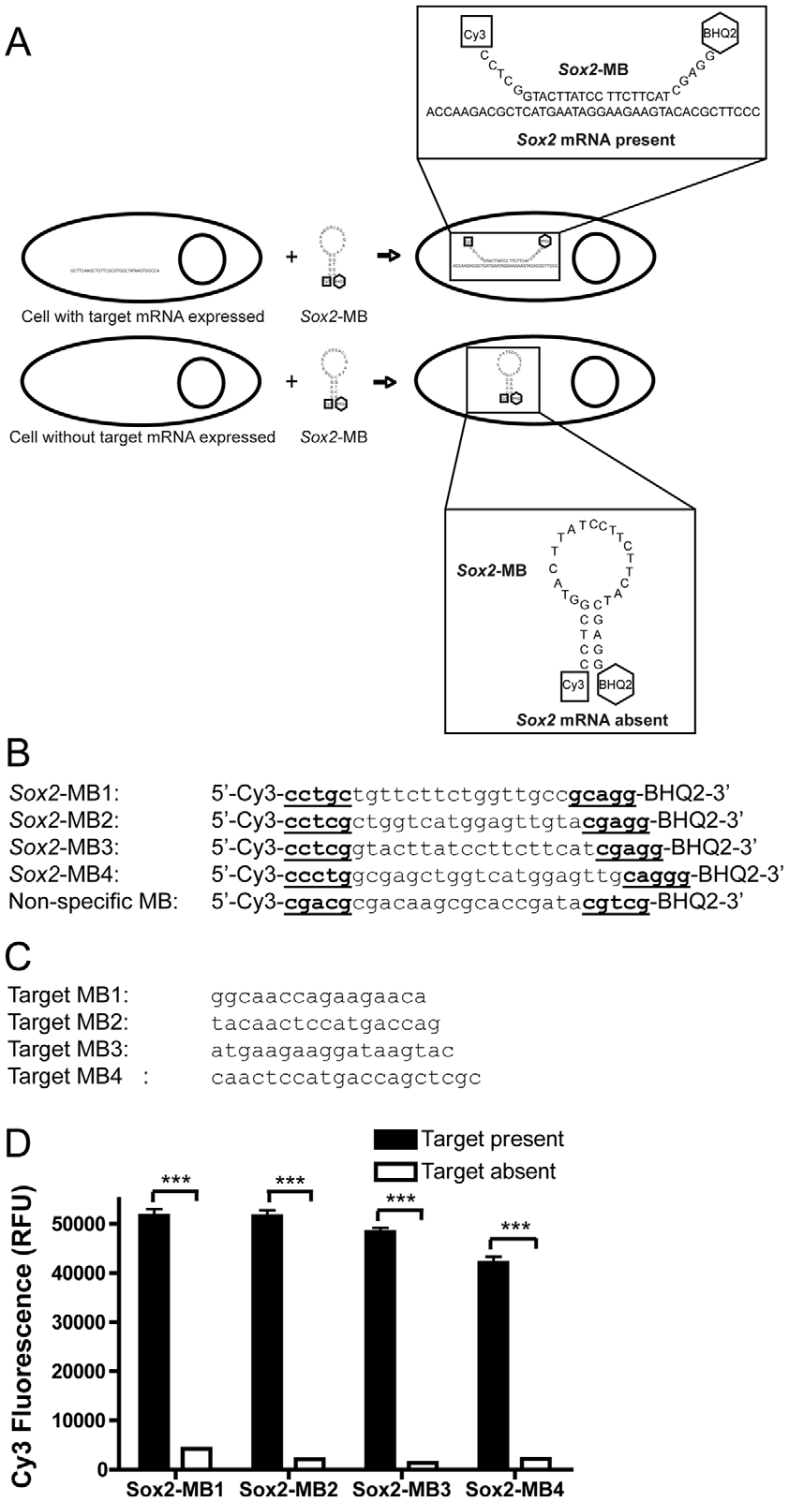
Mechanism and design of *Sox2* mRNA-specific MB. (A) Opening of the *Sox2*-MB is induced in the cytoplasm of cells expressing *Sox2* and emission of Cy3 fluorescence is detected. In contrast, *Sox2*-MB remains in the hairpin conformation in the cytoplasm of *Sox2* negative cells, and no emission of Cy3 fluorescence is detected. (B) The sequences of the designed *Sox2*-MBs and the non-specific-MB. (C) The sequences of the synthesized oligonucleotides complementary to the loop sequence of each *Sox2*-MB. (D) The *Sox2*-MBs were mixed with or without its target sequence and the Cy3-fluorescence was detected with microplate reader. A difference was seen in all of the designed *Sox2*-MBs between when the target sequence was present or not. Error bars represent the mean ± SEM. Asterisks denote statistical significance (n = 3 samples, ^***^ *p*<0.001).

The use of molecular beacons (MBs) as reporters for the presence of mRNA presents a method to sort stem cell populations based on their mRNA expression levels. All expressed mRNAs can be potential targets and thus could be used as sorting parameters. MBs consist of sequences of 25–30 bases in length with a fluorophore attached to the 5′-end and a quencher molecule to the 3′-end [4]. At 37°C, the MB forms a hairpin structure that causes the fluorophore to be quenched. The sequence within the loop of the hairpin is designed to be complementary to the target mRNA of interest (Figure 1A). Upon hybridization of the central loop of the MB to its target, the hairpin opens, correspondingly releasing the fluorophore from the quencher. Therefore, the MB reports only upon binding with the target mRNA.

To explore the use of MBs in live cell sorting of stem cells from mixed populations, we targeted SRY (sex determining region Y)-box2 (*Sox2),* a gene encoding a transcription factor reflective of stemness in embryonic stem cells [5], [6], induced pluripotent stem cells [7] and adult stem cells [8]. We designed and characterized four candidate *Sox2*-targeting MBs. We showed that we could deliver our MBs intracellularly using a simple PEI-based polymer micelle delivery method as well as a commercial method using cationic lipids. Finally, we verified that MBs enable FACS discrimination and sorting of live *Sox2*+ embryonic and somatic stem cells from mixed populations; a capability that should be useful in a wealth of applications.

## Materials and Methods

### Ethics statement

The primary mouse tissue was obtained under ethical approval by the Office Vétérinaire Cantonale Vaud (Switzerland).

### Reagents and cell culture

Cell culture media were from Gibco Invitrogen, and all other reagents were from Sigma-Aldrich unless otherwise stated. R1 mouse embryonic stem (mES) cell lines were purchased from ATCC, and R1 lines expressing green fluorescence protein (GFP) corresponding with Oct4 expression, were kindly donated by Peter Zandstra, University of Toronto [2]. Culture of mES cells was performed as previously described [9]. Briefly, undifferentiated mES cells were maintained on mitomycin C-treated mouse embryonic fibroblasts (MEFs) in standard mES cell culture medium containing leukemia inhibitory factor (LIF; Chemicon International). Differentiation of mES cells was conducted by incubating mES cells for 4 days in Dulbecco's modified Eagle's medium (DMEM) supplemented with 10% fetal bovine serum (FBS; HyClone), 2 mM L-glutamine, 1% (v/v) penicillin streptomycin and 1 µM retinoic acid (RA).

Isolation and culture of mouse neural stem/progenitor cells (mNSCs) were performed as previously described [10], [11]. Briefly, the two neurogenic areas (subventricular zone (SVZ) and hippocampus) of 2–5 days old mice (C57BL6) were dissociated in 300 µL papain:ovomucoid (1∶1) mixture at 37°C for 45 min. The papain mixture consisted of DMEM-F12 containing 30 U/µL papain, 240 µg/mL cysteine and 40 µg/mL DNAseI, and the ovomucoid mix consisted of L15 medium (Sigma-Aldrich) containing 1.125 mg/mL trypsin inhibitor, 0.5 mg/mL bovine serum albumin (BSA) and 40 ng/mL DNAse I. Papain activity was then blocked by addition of one extra volume of ovomucoid mix. Subsequently, the cell suspension was centrifuged (5 min, 80×g). The cell pellet was resuspended in 0.3 mg/mL sucrose and centrifuged for 10 min at 850×g to clear from myelin debris, after which cells were resuspended and cultured in suspension in the standard neurosphere medium (DMEM/F12+ Glutamax) containing 20 ng/mL epidermal growth factor (Peprotech) and B27 supplement for 4 d at 37°C. Neurospheres were passaged with 0.05% trypsin in Versene (Invitrogen) followed by mild mechanical trituration.

### Delivery vehicle

A poly(ethylene imine) (PEI)-based cationic polymer micelle delivery vehicle produced in our lab [12] was used to deliver the MBs into the cytoplasm. Briefly, the cationic micelle vehicle consisted of a diblock copolymer, poly(ethylene glycol)-*bl*-poly(propylene sulfide) (PEG-PPS) and a triblock copolymer, poly(ethylene glycol)-*bl*-poly(propylene sulfide)-*bl*-poly(ethylene imine) (PEG-PPS-PEI). In a volume of 300 µL dichloromethane, 10 mg of diblock copolymer was dissolved together with 1 mg of the triblock copolymer. At room temperature (RT), this solution was dropped into 1 mL of ultrapure water. The solution was then stirred at RT until the organic solvent was completely removed. Subsequently, the aqueous phase containing the formed copolymer blend cationic micelles was mixed with the MBs (see below).

### MB design and synthesis

Four *Sox2* mRNA-specific candidate molecular beacons (Figure S1A) were designed using software that predicts RNA secondary structures (mFOLD, http://www.bioinfo.rpi.edu/applications/mfold/ [13], [14]). The complete murine *Sox2* mRNA was analyzed for potential openings or voids in the mRNA. The target sequences were BLASTed against the mouse genome to ensure specificity to *Sox2* mRNA. The candidate MBs had a Cy3-molecule attached to the 5′-end and a blackhole quencher-2 attached to the 3′-end (Microsynth) (Figure 1A and 1B). A nonspecific-MB target sequence that is not complementary to any known mRNA in mouse was used as a negative control (5′ Cy3-CGAGGCGACAAGCGCACCGATACGTCG-BHQ2 3′ [15]). The four designed *Sox2*-targeted candidate MBs were assayed for fluorescence levels in the presence and the absence of their complementary designed oligonucleotides to their loop sequences (Figure S1B), mixing 0.4 µM MBs with 1 µM oligonucleotide in a 96-well plate. After 1 h of incubation at 37°C, fluorescence was measured at the Cy3 wavelengths (excitation 550 nm/emission 570 nm) using a microplate reader (Saphire^2^; Tecan).

### MB delivery to cells

**Figure 2:**
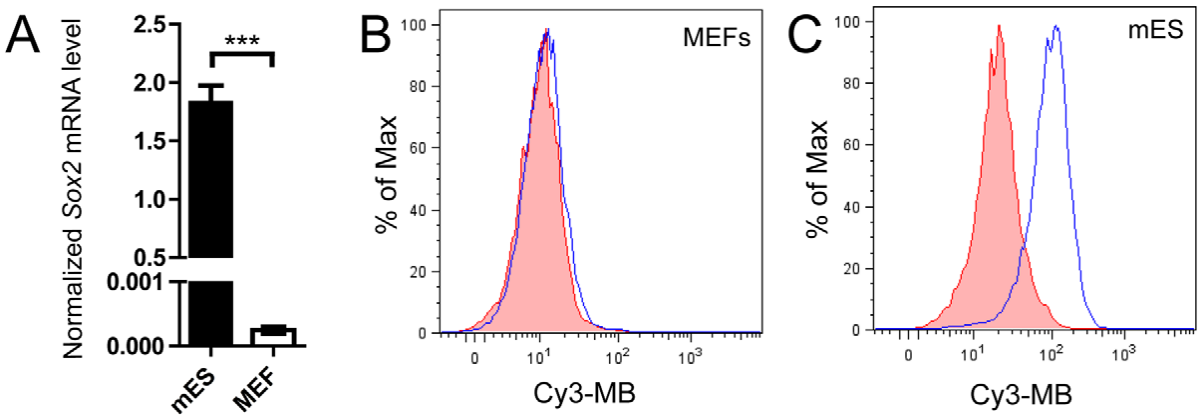
Detection of *Sox2*-MB in undifferentiated mES cells as compared to *Sox2*-negative MEFs. (A) *Sox2* expression in mES cells and MEFs was analyzed by RT-PCR. Fluorescent signals of (B) MEFs and (C) mES cells treated with *Sox2*-MB (blue line) and nonspecific-MB (control, red line) as measured by flow cytometry. Error bars represent the mean ± SEM. Asterisks denotes statistical significance (n = 3 samples,^***^ *p*<0.001).

100 nM *Sox2*-targeting candidate MBs or nonspecific-MB was mixed together with 1 µL of cationic micelles (containing 10 µg of diblock copolymer mixed with 1 µg of triblock copolymer) and incubated at RT for 20 min. Subsequently, the candidate MB solutions were re-suspended in a total volume of 200 µL standard mES cell culture medium containing LIF and added to mES cells grown in 24 well plates. The cells were then incubated at 37°C for 1 h. Alternatively, mNSCs, grown in suspension, were treated with the candidate MBs after centrifugation (3 min, 80×g), as described above but in the standard neurosphere medium. As an alternative method using commercial reagents, lipofectamine-2000 delivery of MBs was done according to the manufacture's protocol (24 well plate DNA transfection, Invitrogen). Briefly, 200 nM *Sox2*-targeted candidate MBs or nonspecific-MB was mixed together with 1 µL lipofectamine-2000 in Opti-MEM (Gibco Invitrogen) and incubated at RT for 20 min. The candidate MB solutions were re-suspended in a total volume of 400 µL DMEM and added to the mES cells. The cells were then incubated at 37°C for 1 h. After incubation, cells were washed twice with D-PBS (Gibco Invitrogen), and respective cell culture medium was added. Fluorescence images were taken with an Axiovert 200 M microscope (Zeiss) or a LSM 700 confocal laser-scanning microscope (Zeiss). Dissociated mES cells were also washed once in D-PBS and were analyzed by flow cytometry using a CyAN ADPS (Beckman Coulter). Analysis was done with FlowJo software (Tree Star) (Figure 2C).

### Immunostaining

To stain for stemness markers, cells were permeabilized with 0.4% saponin (Applichem) in D-PBS for 30 min. After blocking for 1 h (3% BSA and 0.4% saponin in D-PBS), the cells were incubated with primary antibodies for 2 h at RT. Primary antibodies used were anti-SSEA1 (mab4301, Chemicon), anti-Sox2 (48–1400, Invitrogen), anti-Nestin (611658, BD Bioscience) and anti-Nanog (ab80892, Abcam). After washing in D-PBS, cells were incubated for 2 h with secondary mouse antibody conjugated to Alexa Fluor 488 and secondary rabbit antibody conjugated to Alexa Fluor 546 (Invitrogen). After washing, Hoechst 33342 (Invitrogen) was added to the cells and incubated for 10 min before imaging with an Axiovert 200 M microscope (Zeiss).

### Real-time PCR

mRNA was isolated using a RNeasy Plus Mini Kit (Qiagen) according to the manufacturer's instruction, and the extracted mRNA concentration was measured with NanoDrop^TM^ 1000 spectrophotomer (Thermo Fisher Scientific). An amount of 1 µg mRNA was used to produce cDNA with the iScript cDNA Synthesis kit (Bio-Rad Laboratories) and analysis of mRNA level were performed by the iQ SYBR Green Supermix (Bio-Rad Laboratories). Standard curves for each primer were plotted and samples were measured in triplicate with an iCycleriQ Multicolor Realtime PCR detection system (Bio-Rad Laboratories). The mRNA levels of genes were normalized to that of a housekeeping gene, beta-actin. General information and sequences of primer designed with cDNA sequences obtained from GenBank for mouse and by Primer3 software (Whitehead Institute/MIT Center for Genome Research) (Table S1).

### Flow cytometry, cell sorting and analysis

**Figure 3:**
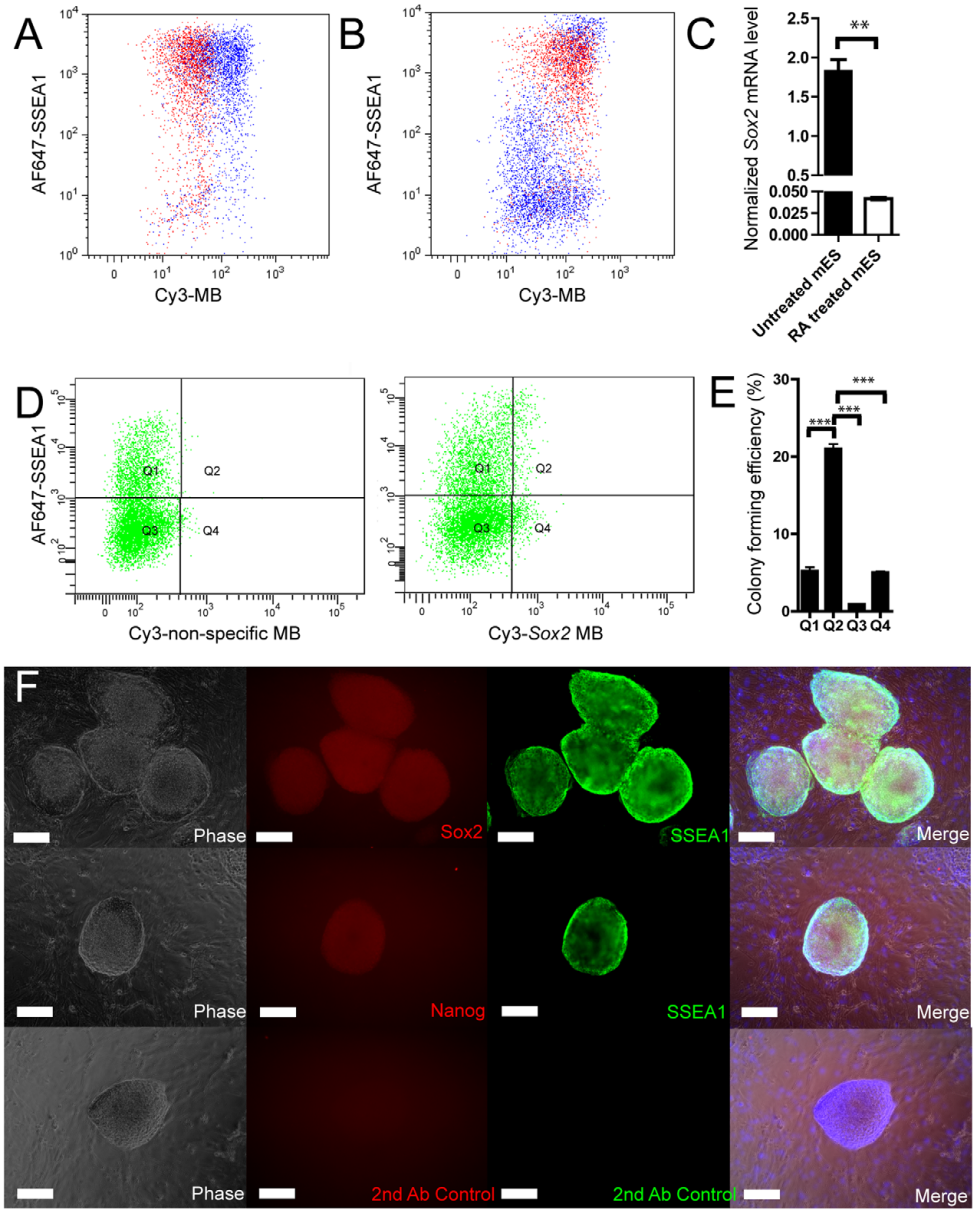
Detection of *Sox2*-MB in differentiated mES cells. (A) mES cells stained for SSEA-1 together with the *Sox2*-MB (blue dots) and the nonspecific-MB (red dots). (B) SSEA-1 stained differentiated mES cells treated with *Sox2-*MB (blue dots) were compared to SSEA1 stained undifferentiated mES treated *Sox2-*MB (red dots). (C) Undifferentiated mES cells and mES cells differentiated by exposure to RA were analyzed with RT-PCR. (D) Four quadrants (Q1, Q2, Q3 and Q4) of the differentiated mES cells were selected by comparing the nonspecific-MB fluorescent signal with the *Sox2*-MB fluorescent signal. (E) The double-positive sorted cell populations (Q2: Sox2-MB^+^ and SSEA1^+^) formed significantly more undifferentiated colonies compared to the positive-negative sorted cell populations (Q1: Sox2-MB^-^ and SSEA1^+^ Q4: Sox2-MB^+^ and SSEA1^−^ ), and the double-negative sorted cell population (Q3: Sox2-MB^-^ and SSEA1^−^). (F) Undifferentiated colonies were positively stained for Sox2, Nanog and SSEA1 (Scale bar  = 200 µm). Error bars represent the mean ± SEM. Asterisks denotes statistical significance (n = 3 samples ^**^ *p*<0.01, n = 4 samples^***^ *p*<0.001).

mES cells treated with RA were used for analysis and sorting. Dissociated cells were re-suspended in D-PBS (Gibco Invitrogen) and filtered through a 70 µm cell strainer (BD-Falcon). Cells were treated with MBs as described above. Then, cells were incubated for 15 min in Alexa Fluor 647 SSEA-1 antibody (51–8813, eBioscience), were washed once in D-PBS and were analyzed by flow cytometry using a CyAN ADPS (Beckman Coulter). Analysis was done with FlowJo software (Tree Star). mES cells were sorted using a FACSVantage (BD Bioscience) into a 24-well plate. The nonspecific-MB was used to set the quadrants in the dot-plot of SSEA1 expression versus MB signal. From each quadrant, SSEA1^+^/*Sox2-*MB^−^ (Q1), SSEA1^+^/*Sox2*-MB^+^ (Q2), SSEA1^−^/*Sox2*-MB^−^ (Q3) and SSEA1^+^/*Sox2*-MB^−^ (Q4), 500 cells were sorted and cultured for 5 d (Figure 3D). Subsequently, colonies of mES cells were fixed with 10% (v/v) natural buffered formalin, and undifferentiated colonies were counted to calculate the colony forming efficiency by dividing with the initial sorted number of cells.

**Figure 4:**
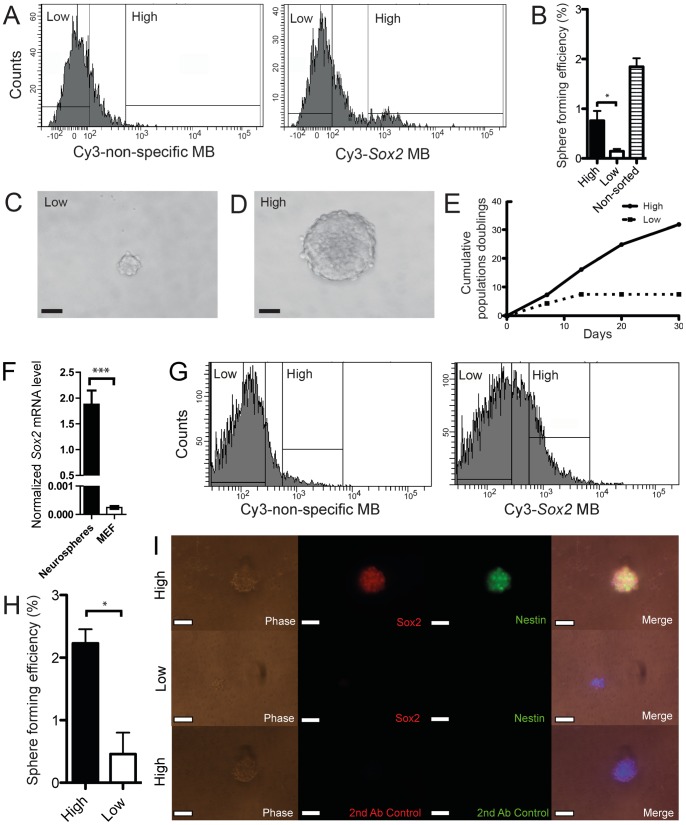
Isolation of neurospheres from primary mouse tissue and of *in vitro* cultured neurospheres using *Sox2-*MB. (A) Two cell populations, namely *Sox2-*MB^high^ and the *Sox2-*MB^low^, were first selected on Annexin-V^-^ cells and then by comparing the nonspecific-MB fluorescent signal to the Sox2-MB fluorescent signal. (B) After 1 wk, sphere-forming efficiency was calculated from the *Sox2-*MB^high^ and the *Sox2-*MB^low^ populations as well as non-sorted primary mouse hippocampus isolated cells. (C and D) Images of 1 wk old spheres generated from sorted *Sox2-*MB^low^ cells and *Sox2-*MB^high^ cells (scale bar  = 25 µm). (E) Neurospheres from the *Sox2-*MB^high^ and the *Sox2-*MB^low^ populations were serially passaged and cumulative population doublings was calculated. (F) *In vitro* cultured neurosphere mRNA expression of *Sox2* was analyzed by RT-PCR and compared to MEFs. (G) Two cell populations, namely *Sox2*-MB^high^ and *Sox2-*MB^low^, were selected by comparing the nonspecific-MB fluorescent signal to the *Sox2-*MB fluorescent signal. (H) After 1 wk, sphere-forming efficiencies were calculated. (I) Neurospheres formed by the *Sox2-*MB^high^ and the *Sox2-*MB^low^ populations were stained for Sox2 and Nestin, or secondary antibodies only (control) (scale bar  = 50 µm). Error bars represent the mean ± SEM. Asterisks denotes statistical significance ((n = 5 samples ^*^ *p*<0.05, n = 3 samples^***^ *p*<0.001).

Primary isolated mNSC or cultured neurospheres were dissociated in single cell suspension and treated with the nonspecific-MB to set the sorting gate for a high and low population of neurospheres. The *Sox2*-MB-treated primary isolated mNSC or cultured neurospheres were sorted into a *Sox2-*MB^high^ and *Sox2-*MB^low^ population. 350 cells in triplicate were plated into a 96-well plate using a FACSAria II (BD Bioscience). The sorted cells were either fixed with 10% natural buffered formalin after 1 wk of culture and imaged (Inverted motorized IX81 microscope, Olympus) or continued to be serially passaged. Sphere forming efficiency was calculated by manually counting all the spheres and then divided with the initial number of sorted cells. Population doublings (PD) was calculated using the following formula: PD  =  Log(N/N0)/Log(2), where the N0 is the number of seeded cells and N was the calculated number of cells at the time of passaging using a hemocytometer. 5 minutes before the sort of primary isolated NSCs, 5 µL of Annexin-V-Cy5 (Biovision, LuBioScience) was added to 500 µL of MB treated cells. Annexin-V negative cells were selected prior to setting the gates for *Sox2-*MB^high^ and *Sox2-*MB^low^ populations (Figure 4 A and G).

### Statistical Analysis

The two-tailed unpaired Student's t-test was used to analyze if a difference in two data sets was statistically significant. A p-value of less than 0.05 was considered significant (*p<0.05, **p<0.01 ***p<0.001). All the error bars represent the standard error of the mean (S.E.M.).

## Results

### *Sox2-*MBs detect their targets and discriminate between *Sox2*-positive and *Sox2*-negative cells

Four different MBs targeting Sox2 (*Sox2-*MBs) were designed (Figure 1B). To determine their sensitivity to their complementary target sequences, we measured Cy3 emission from the candidate *Sox2-*MBs *in vitro* in the presence and absence of their targets (Figure 1C and 1D). For all MBs assayed, a difference of 12-fold or more in Cy3 fluorescence was seen between the presence and absence of the complementary sequences, indicating functional molecular beacon reporting for all four candidates.

We then assayed if our *Sox2-*MBs could be used to distinguish between *Sox2*-negative and *Sox2*-positive cell populations (i.e. if the MBs would recognize their targets in the complex milieu *in vivo* within the cell). As a model system to study the activity of our beacon, we choose mES, which are known to express *Sox2*. MEFs were used as negative control. *Sox2* expression was first confirmed by RT-PCR (Figure 2A). MBs were delivered to cells using as a delivery vehicle the cationic micelles, consisting of a hydrophobic core, a hydrophilic corona of poly(ethylene glycol), and a cationic poly(ethylene imine) chain embedded in the corona [12]. As expected, when *Sox2*-negative MEFs were treated with the candidate *Sox2-*MBs or nonspecific-MB and analyzed by flow cytometry, neither showed a fluorescence signal (Figure 2B, Figure S1A). In contrast, when the *Sox2-*MBs were incubated with mES cells, two of the MBs (*Sox2-*MB1 and *Sox2*-MB3) clearly displayed an increase in fluorescent as detected by microscopy (Figure S2), whereas the nonspecific-MB (*Sox2-*MB2 and *Sox2-*MB4) did not show fluorescence over background in both the feeder cultures and the mES colonies. Similar results were obtained by flow cytometry: *Sox2-*MB1 and *Sox2-*MB3 showed a 2.6 and 4.6-fold higher mean fluorescence signal as compared with the nonspecific-MB (Figure 2C, Figure S1B). Based on these results from microscopy and flow cytometry, we selected *Sox2-*MB3 for further study (from hereon referred to simply as *Sox2-*MB; 5′ Cy3-CCTCGGTACTTATCCTTCTTCATCGAGG-BHQ2 3′).

To test if a commercially available delivery vehicle can also be used to deliver the *Sox2*-MB to mES cells we used lipofectamine-2000, a cationic lipid. Flow cytometry showed that the *Sox2-*MB had a 2.0-fold higher mean fluorescence as compared with the nonspecific-MB (Figure S3). The cationic micelle delivery vehicle (Figure 2C) provided a 4.6-fold higher mean fluorescence signal when delivering Sox2-MB in Sox2+ cells than did the cationic lipid vehicle (Figure S3).

To compare our *Sox2*-MB reporter to a commonly used intracellular fluorescent reporter system for mES cells, we made use of a previously described Oct4-GFP reporter cell line [2]. We delivered our *Sox2-*MB to Oct4-GFP mES cells. The *Sox2-*MB co-localized inside GFP-positive cells, as shown by confocal microscopy, confirming reporting of *Sox2*-MB (Figure S4).

Furthermore, to verify that *Sox2-*MB did not influence mRNA expression of stemness genes in mES cells, such as *Nanog* and *Sox2*, RT-PCR was used to measure gene expression levels. Thus, mRNA was isolated from mES cells treated with *Sox2-*MB for 1 h and 24 h. There were no significant differences in the mRNA levels of the stemness genes in *Sox2-*MB-treated mES compared to untreated mES cells (Figure S5).

### *Sox2-*MB mark pluripotent cells and can be used for sorting live mES cells in heterogeneous populations

To confirm that *Sox2*-MB label naïve mES cells and not their committed progeny, cells were stained for the well-known mES cell marker, SSEA1 (stage-specific embryonic antigen-1). As expected, mES cells cultured under self-renewal conditions were double-positive for both the *Sox2-*MB and SSEA1 (Figure 3A). When mES cells were induced to differentiate by incubation with RA, committed cells in these mixed cultures showed a lower fluorescence signals as compared to primitive mES cells (Figure 3B). This was also confirmed by RT-PCR; differentiated mES progeny had significantly lower *Sox2* expression than mES cells (Figure 3C). Interestingly, 20% of mES cells that were treated with RA still maintained positivity for the two markers (SSEA1^+^/*Sox2*-MB^+^), indicating that our 4 day differentiation treatment was only 80% effective (Figure 3B).

To determine if *Sox2-*MB- and SSEA1-double positive mES cell populations would indeed show phenotypic characteristics of pluripotent cells, the SSEA1^+^/*Sox2*-MB^+^ population was FACS sorted, and colony formation was assessed. The double-positive population (Q2, Figure 3D) formed at least 4-fold more mES colonies than the other three populations (Figure 3E). Furthermore, these sorted cells expressed pluripotency markers *Sox2*, *Nanog* and SSEA1 (Figure 3F), confirming that the *Sox2-*MB can be used to sort stem cells from a mixed cell population.

### *Sox2*-MB can be used to sort live *Sox2*-positive cells from neurospheres

To demonstrate that our designed *Sox2*-MB could be used with other stem cells, an additional stem cell type expressing the *Sox2* transcription factor was evaluated. Neural stem and progenitor cells can be isolated and expanded *in vitro* through a commonly used neurosphere assay [3], [8] where epidermal growth factor-responsive cells are selected for their capacity to expand *in vitro* as free floating aggregates. Prospective isolation of NSCs has been previously performed using cell surface markers or transgenic fluorescent reporter lines [16].We tested here the possibility of adopting a mRNA based approach for selection by targeting *Sox2*.

Cells freshly dissected from the two neurogenic areas (SVZ and hippocampus) of 2–5 day old C57BL6 mice were treated with the MBs. *Sox2-*MB-treated cells had a higher fluorescence than cells treated with the nonspecific-MB (Figure 4A). The brightest 1.3% of cells (*Sox2*-MB^high^) were sorted and assayed for their capacity to form neurospheres and compared to cells with low fluorescence (*Sox2*-MB^low^, Figure 4A). *Sox2*-MB^high^ sorted cells generated significantly more neurospheres compared to the *Sox2*-MB^low^ cells, which were also larger in size (Figure 4B, C and D). Moreover, the *Sox2*-MB^high^ sorted neurospheres kept producing neurospheres with passaging in comparison to the *Sox2*-MB^low^ (Figure 4E). Thus, *Sox2*-MB can be used to sort neurosphere-forming cells from primary isolated tissues. Nevertheless, we did not generate a greater number of neurospheres by culture of *Sox2*-MB-based-sorted cells than by culture of non-sorted freshly isolated cells (Figure 4C).

Cells that were expanded for several passages *in vitro* maintained *Sox2* expression, as shown by RT-PCR (Figure 4F). When neurospheres were treated with the MBs, *Sox2-*MB-treated cells had 1.9-fold higher fluorescence than cells treated with the nonspecific-MB (Figure 4G). Also in this case, *Sox2*-MB^high^ sorted cells formed more neurospheres that were also significantly larger (>50 µm) than the *Sox2*-MB^low^ sorted cells (Figure 4H). *Sox2*-MB^high^ sorted cells also expressed mNSC markers Nestin and Sox2, as shown by microscopy (Figure 4I).

## Discussion

We show the identification and characterization of a *Sox2*-targeting MB that can be delivered by chemical means to cells and used in live-cell-sorting of multiple cell types. Furthermore, *Sox2*-MB-based sorting allowed recovery of undifferentiated mES cells from a pool of RA-differentiated mES cells in which 80% of the cells had differentiated, and it allowed isolation and enrichment of neurosphere-forming cells based on the intensity of *Sox2*-MB reporting. As such, *Sox2*-MB appear to be useful for both positive and negative selections of stem cells from mixed populations. Importantly, we demonstrated that while the *Sox2-*MB binds to mRNA and fluoresces in the cytoplasm of cells expressing *Sox2*, binding does not influence expression of stemness genes in the treated cells.

Other research groups have used MBs to target various mRNAs related to stemness, including *survivin* [17], *Bmp4* [15] and *Oct4* [18]. Although these groups reported that they could detect specific MB signals by either fluorescence microscopy or flow cytometry, they did not demonstrate that designed MBs could be used for live cell sorting purposes. A more recent publication, however, described the post-sorting effects on cells sorted with a dual-FRET molecular beacon targeting *Oct4* [19], using electroporation to deliver *Oct4*-MB to human embryonic stem (hES) cells. The positive-sorted cells showed properties of hES cells *in vitro* and *in vivo*. Here, we show that the *Sox2-*MB can be delivered with a cationic micelle vehicle or a cationic lipid vehicle to mES cells and mNSCs in both positive and negative sorting. The demonstration of live cell sorting raises the possibility of direct sorting of rare adult stem cells from primary cell isolates from tissues, to greatly accelerate the process of tissue-specific stem cell derivation.

## Supporting Information

Figure S1 **FACS analysis of MEFs and mES cells treated with** ***Sox2-*** **MBs and nonspecific-MB.** (A) MEFs and (B) mES cells were treated with *Sox2-*MB1 (blue line), *Sox2-*MB2 (green line), *Sox2-*MB3 (orange line), *Sox2-*MB4 (cyan line) and nonspecific-MB (red line).
(TIF)

Figure S2 **Microscopy of living mES cells treated with** ***Sox2-*** **MBs with phase and fluorescent images.** mES cells were treated with (A,B) *Sox2-*MB1, (C,D) *Sox2-*MB2, (E,F) *Sox2-*MB3, (G,H) *Sox2-*MB4 and (I,J) nonspecific-MB. Scale bar  = 200 µm.
(TIF)

Figure S3 ***Sox2*** **-MB delivered with Lipofectamine-2000 to mES cells.** Fluorescent signals of mES cells treated with *Sox2*-MB (blue line) and nonspecific-MB (control, red line) as measured by flow cytometry.
(TIF)

Figure S4 **Confocal microscopy of Oct4-GFP mES cells treated with** ***Sox2*** **-MB.** (A) Living Oct4-GFP mES cells treated with the *Sox2-*MB with orthogonal slices in the xz-plane and yz-plane are shown. (B) As a control, living Oct4-GFP mES cells treated with the nonspecific-MB with orthogonal slices in the xz-plane and yz-plane are shown. Scale bar  = 20 µm.
(TIF)

Figure S5 **The effect of** ***Sox2-*** **MB on the mRNA level of stemness genes on treated and untreated mES cells.** Cells were analyzed for (A) *Sox2* and (B) *Nanog* mRNA expression after 1 h and 24 h of treatment with the *Sox2-*MB. As controls, untreated mES cells were analyzed in parallel. (n = 4 per sample, ns = not significant) Error bars represent the mean ± SEM.
(TIF)

Table S1 **Primers used for Real-time PCR.**
(TIF)
