# Supplementary figures and images for: Sorting Live Stem Cells Based on Sox2 mRNA Expression
Source: PLoS One. 2012 Nov 27;7(11):e49874. doi: 10.1371/journal.pone.0049874 (PMC3507951; doi:10.1371/journal.pone.0049874)

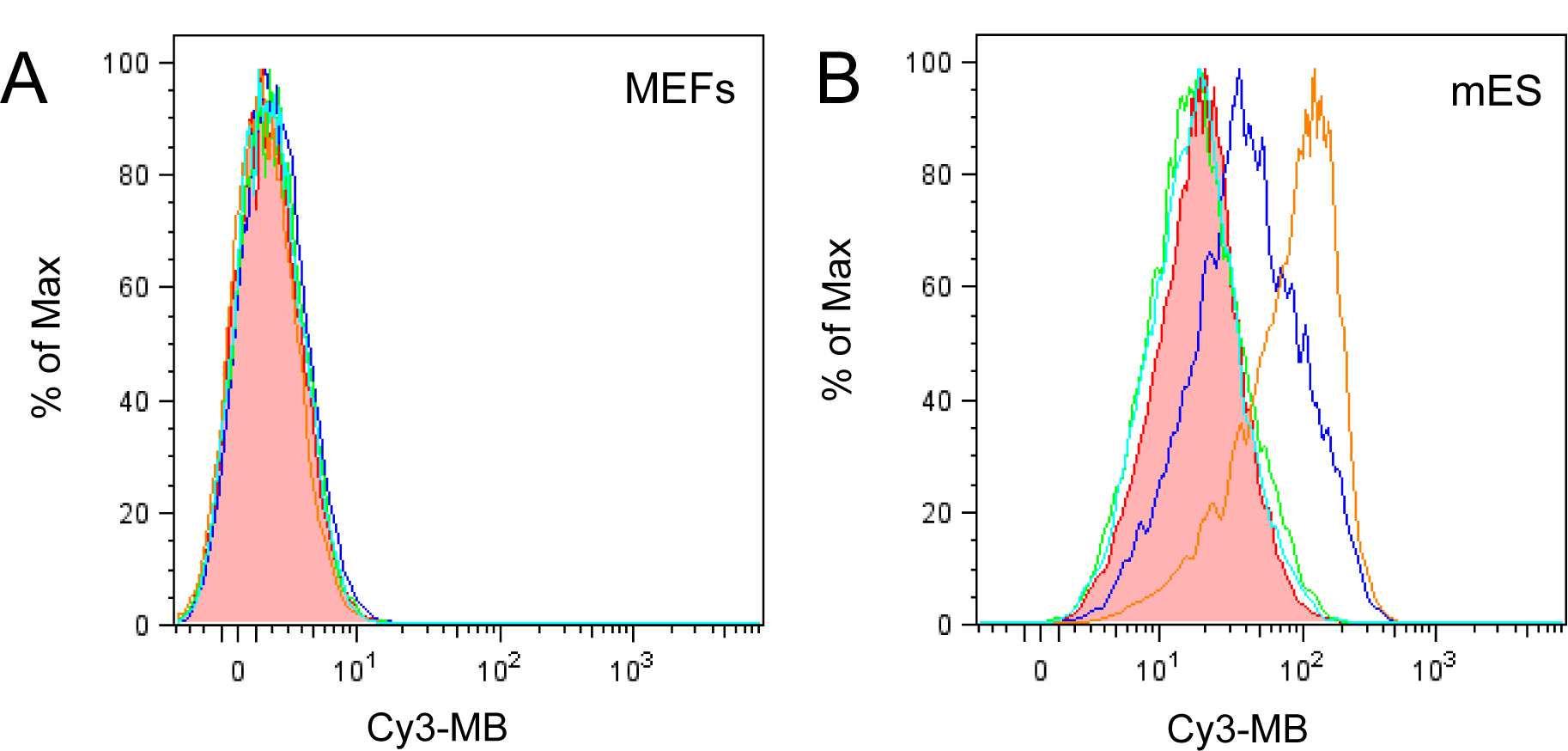

Supplement: Figure S1 — FACS analysis of MEFs and mES cells treated with Sox2- MBs and nonspecific-MB. (A) MEFs and (B) mES cells were treated with Sox2-MB1 (blue line), Sox2-MB2 (green line), Sox2-MB3 (orange line), Sox2-MB4 (cyan line) and nonspecific-MB (red line). (TIF) [file pone.0049874.s001.tif]

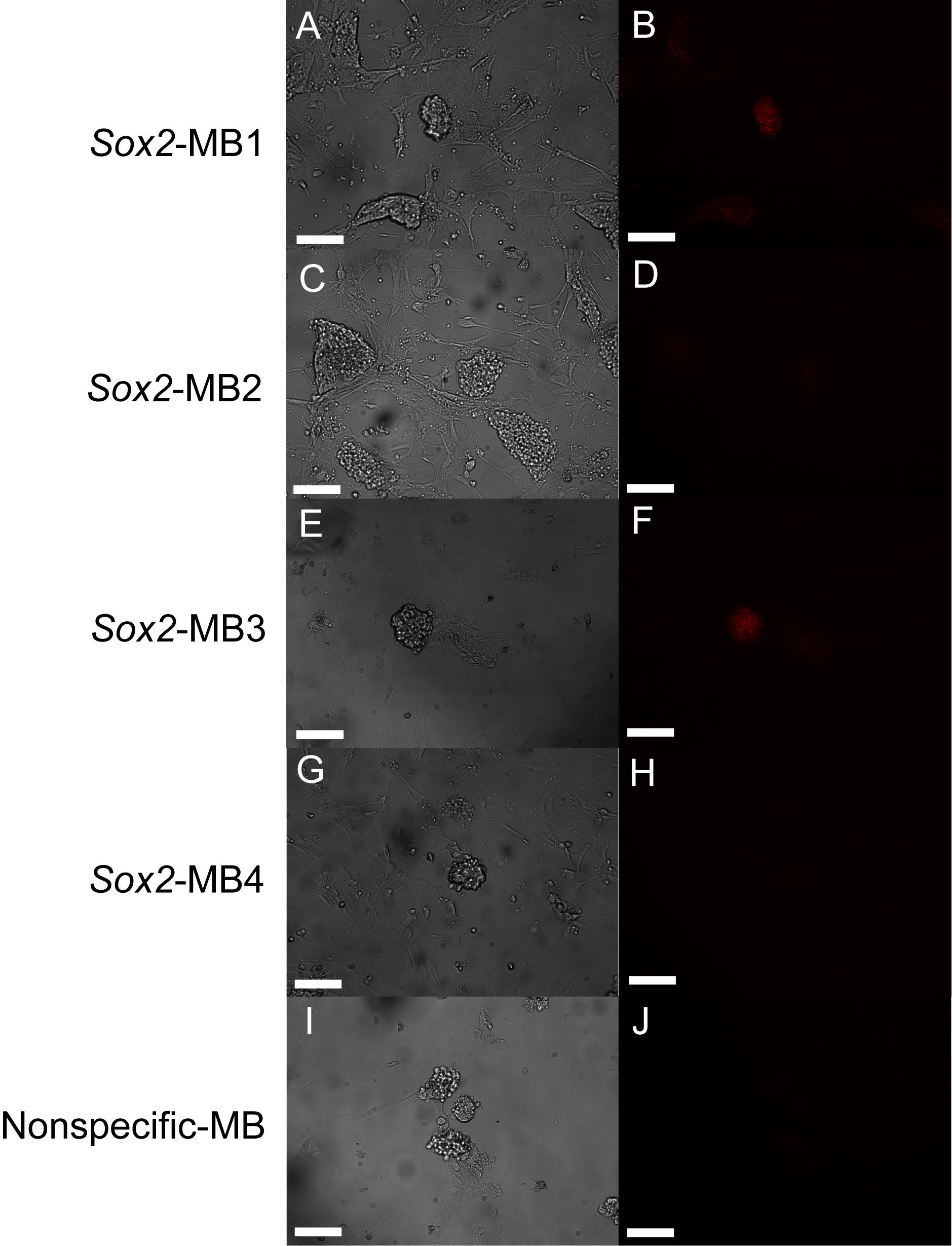

Supplement: Figure S2 — Microscopy of living mES cells treated with Sox2- MBs with phase and fluorescent images. mES cells were treated with (A,B) Sox2-MB1, (C,D) Sox2-MB2, (E,F) Sox2-MB3, (G,H) Sox2-MB4 and (I,J) nonspecific-MB. Scale bar = 200 µm. (TIF) [file pone.0049874.s002.tif]

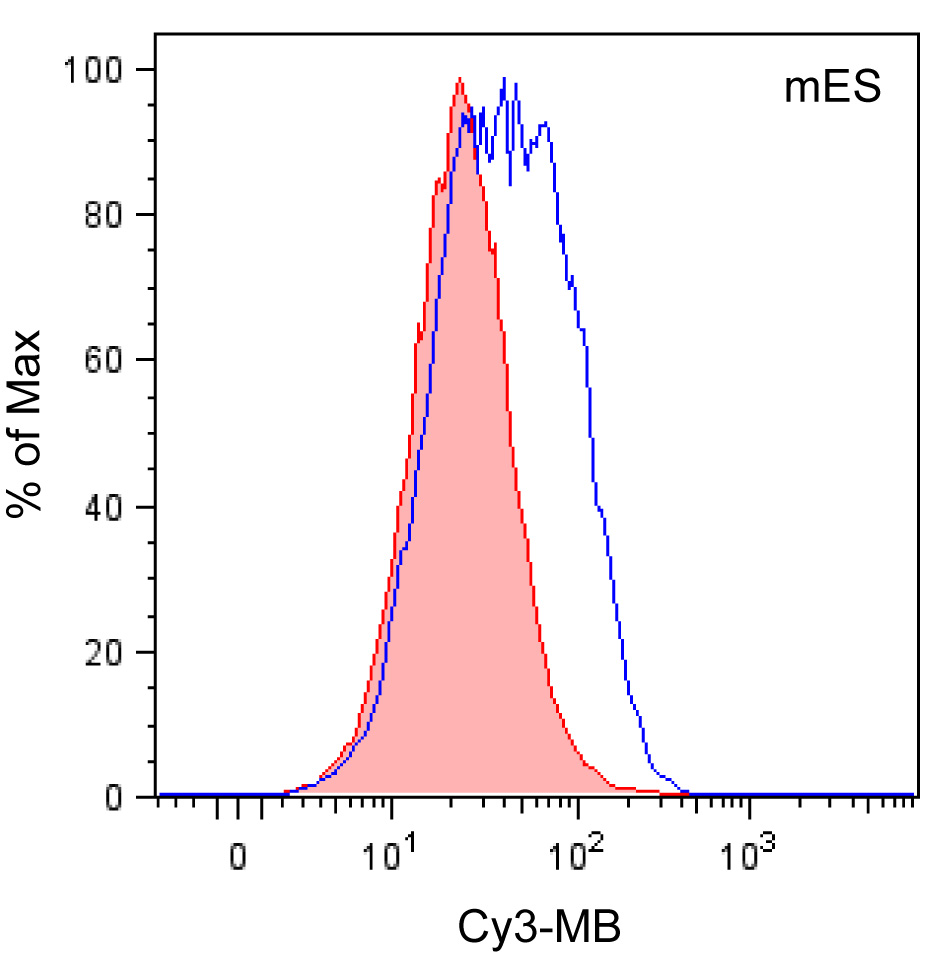

Supplement: Figure S3 — Sox2 -MB delivered with Lipofectamine-2000 to mES cells. Fluorescent signals of mES cells treated with Sox2-MB (blue line) and nonspecific-MB (control, red line) as measured by flow cytometry. (TIF) [file pone.0049874.s003.tif]

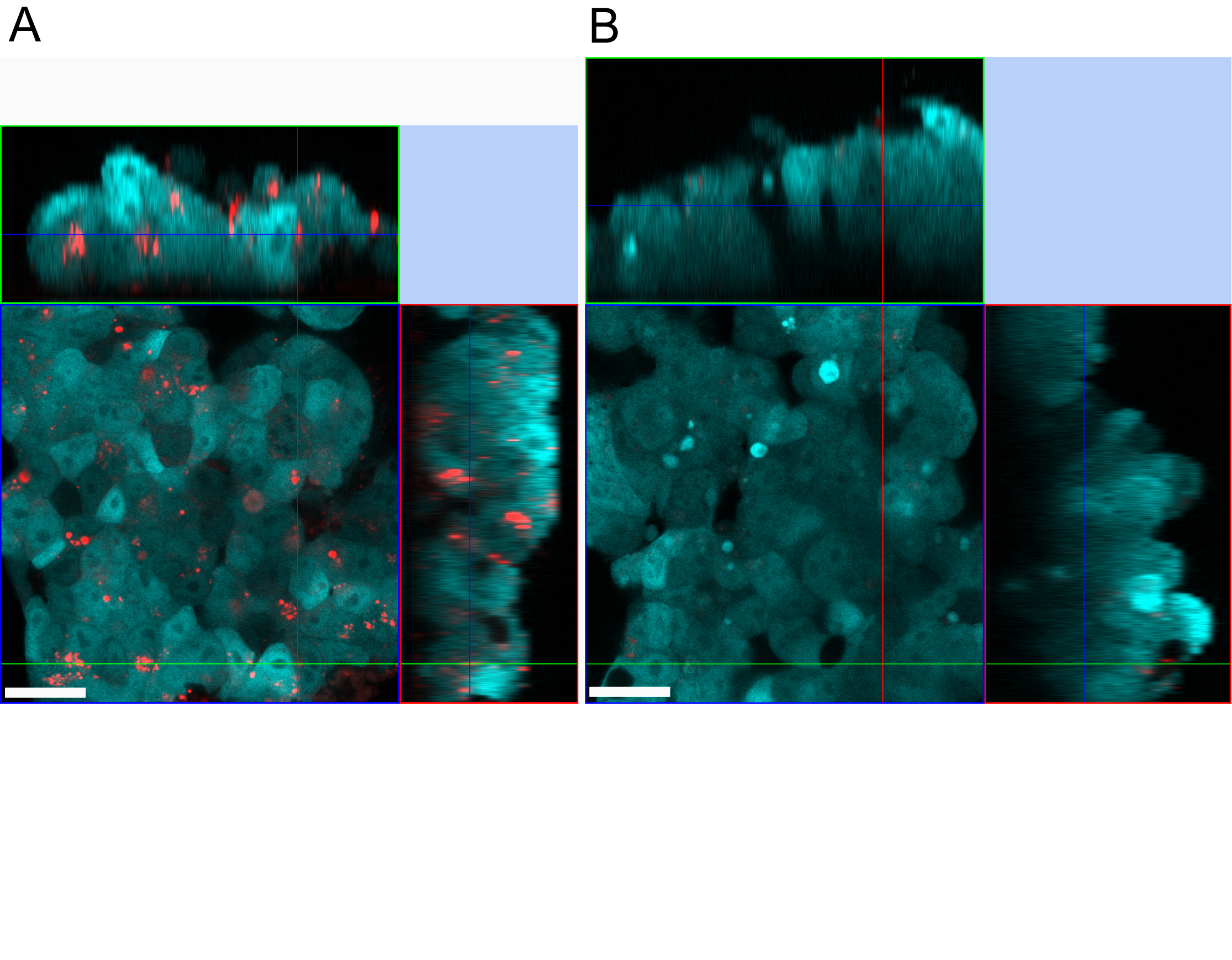

Supplement: Figure S4 — Confocal microscopy of Oct4-GFP mES cells treated with Sox2 -MB. (A) Living Oct4-GFP mES cells treated with the Sox2-MB with orthogonal slices in the xz-plane and yz-plane are shown. (B) As a control, living Oct4-GFP mES cells treated with the nonspecific-MB with orthogonal slices in the xz-plane and yz-plane are shown. Scale bar = 20 µm. (TIF) [file pone.0049874.s004.tif]

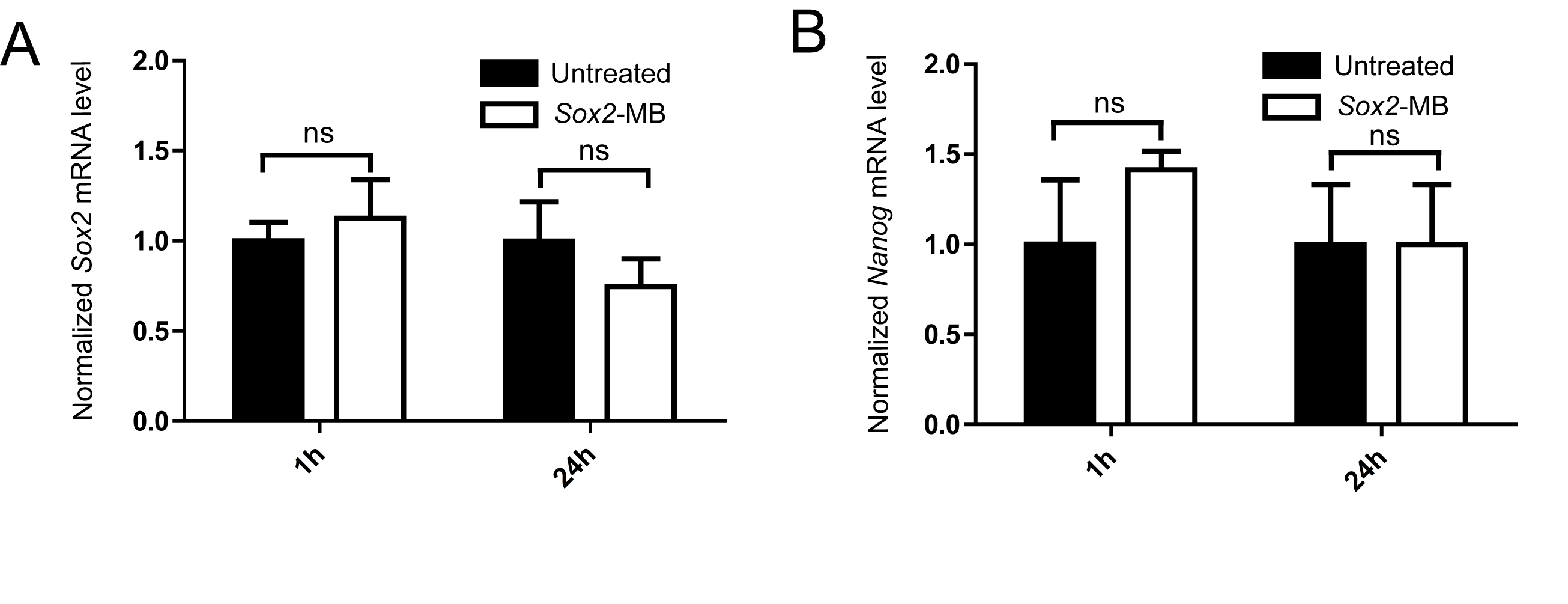

Supplement: Figure S5 — The effect of Sox2- MB on the mRNA level of stemness genes on treated and untreated mES cells. Cells were analyzed for (A) Sox2 and (B) Nanog mRNA expression after 1 h and 24 h of treatment with the Sox2-MB. As controls, untreated mES cells were analyzed in parallel. (n = 4 per sample, ns = not significant) Error bars represent the mean ± SEM. (TIF) [file pone.0049874.s005.tif]

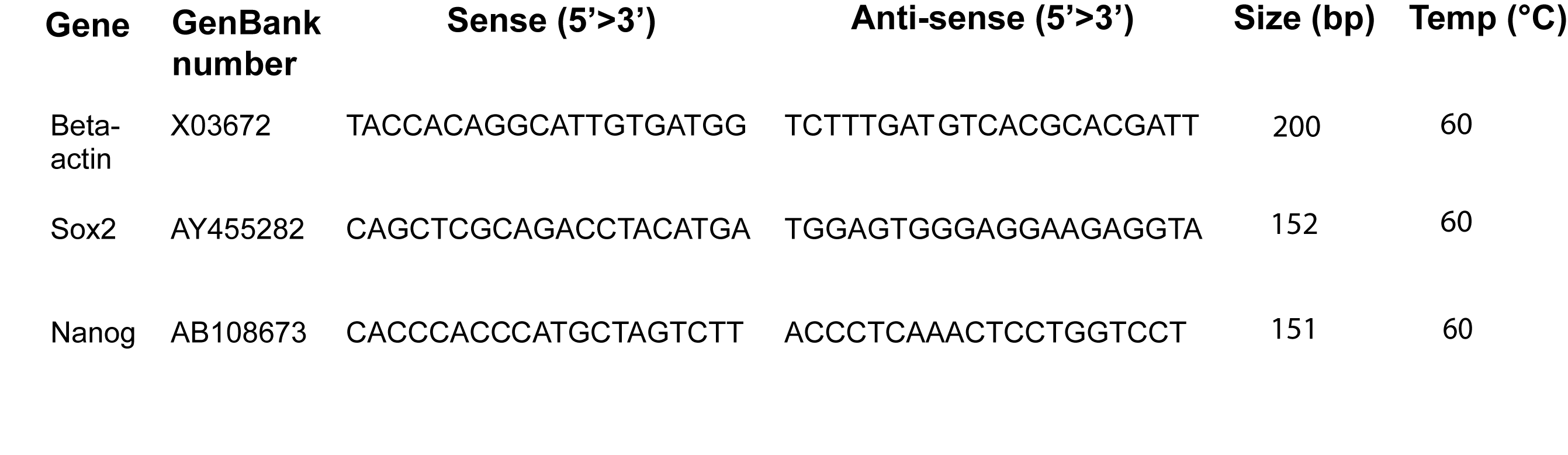

Supplement: Table S1 — Primers used for Real-time PCR. (TIF) [file pone.0049874.s006.tif]
